# A framework for quantifying uncertainty in DFT energy corrections

**DOI:** 10.1038/s41598-021-94550-5

**Published:** 2021-07-29

**Authors:** Amanda Wang, Ryan Kingsbury, Matthew McDermott, Matthew Horton, Anubhav Jain, Shyue Ping Ong, Shyam Dwaraknath, Kristin A. Persson

**Affiliations:** 1 Department of Materials Science and Engineering, University of California, Berkeley, CA 94720 USA; 2 Materials Sciences Division, Lawrence Berkeley National Laboratory, 1 Cyclotron Road, Berkeley, CA 94720 USA; 3 University of California San Diego, La Jolla, CA 92093 USA; 4 Molecular Foundry, Lawrence Berkeley National Laboratory, 1 Cyclotron Road, Berkeley, CA 94720 USA

**Keywords:** Computational methods, Density functional theory, Materials science, Theory and computation, Electronic structure

## Abstract

In this work, we demonstrate a method to quantify uncertainty in corrections to density functional theory (DFT) energies based on empirical results. Such corrections are commonly used to improve the accuracy of computational enthalpies of formation, phase stability predictions, and other energy-derived properties, for example. We incorporate this method into a new DFT energy correction scheme comprising a mixture of oxidation-state and composition-dependent corrections and show that many chemical systems contain unstable polymorphs that may actually be predicted stable when uncertainty is taken into account. We then illustrate how these uncertainties can be used to estimate the probability that a compound is stable on a compositional phase diagram, thus enabling better-informed assessments of compound stability.

## Introduction

Accurate first-principles calculations of phase equilibria are essential for rapid screening of new materials in a variety of technological domains, such as energy storage electrodes, structural metal alloys, semiconductors, catalysts, and CO$_2$ storage materials^1–6^. Density functional theory (DFT) is the most widely used computational method for calculating solid-state phase stability, with many functional approximations and numerous simulation codes developed over the past thirty years^1,7–9^. Although DFT itself is an exact theory for computing ground-state energies, approximate DFT functionals such as the popular Perdew–Burke–Ernzerhof (PBE) functional^10^ result in systematic errors, especially for diatomic gases^11,12^ and transition metal compounds with localized electronic states^12–14^. As a result, DFT-computed formation enthalpies for compounds involving these elements can exhibit errors of several hundred meV/atom^14–17^. Errors in solid-phase reaction enthalpies that do not directly involve elements are typically smaller due to cancellation of errors, but may still differ from experimental values by tens of meV/atom^18^. Such errors impede accurate prediction of phase equilibria and other energy-related properties from first-principles calculations.

DFT errors in solid state thermochemistry have historically been considered to arise from electron self-interaction in compounds with localized electronic states which results in systematic errors in the DFT description of certain anions^14,19^. Self-interaction error in cations is usually mitigated by applying a Hubbard *U* to the *d* or *f* orbitals^14,20^, while various schemes have been developed to mitigate the error associated with anions by applying energy corrections to specific elements, oxidation states, and/or bonds. Wang et al.^12^ fit a constant energy correction to the O$_2$ molecule, and this method was later applied to other diatomic gases (H$_2$, Cl$_2$, F$_2$, N$_2$)^11^. Later correction schemes addressed both anion and self-interaction error by combining constant energy corrections with Hubbard *U* values. For example, the GGA/GGA+*U* mixing scheme of Jain et al.^14^ computed transition metal oxides and fluorides in GGA+*U* using a separately-fit *U* value for each transition metal, then applied energy corrections to both diatomic gases and transition metals to ensure compatibility between the GGA and GGA+*U* energies. The Fitted Elemental Reference Energies (FERE) method^21,22^ used a single *U* value for all transition metals, and assigned an energy correction to every element. Other correction schemes incorporated information about the local bonding environment, such as the sulfide/disulfide corrections described by Yu et al.^19^, or more general correction schemes described by Aykol and Wolverton^23^ and the recent “Coordination-Corrected Formation Enthalpy” (CCE) method of Friedrich et al.^16^.

By fitting energy corrections to experimental data, such empirical DFT correction schemes can reduce the mean absolute errors (MAEs) in computed formation enthalpies to 50 meV/atom or less^14,16,22,23^, with the schemes that incorporate information about the local environment generally resulting in the smallest errors. However, the fitted corrections introduce uncertainty due to (1) uncertainty in the underlying experimental data and (2) the sensitivity of the corrections to the amount of data available for fitting. Given the small magnitude of the energy differences that are relevant in phase diagram construction (e.g., almost degenerate polymorphism and unstable phases within a few meV/atom of the energy convex hull^15,24,25^), quantifying these uncertainties would provide important context for interpreting computationally-predicted phase equilibria. However, few attempts have been made to do so. Lany^21^ simulated experimental noise via a Gaussian distribution, while Grindy et al.^11^ analyzed the uncertainty due to the selection of fit parameters. Yu et al.^19^ quantified both types of uncertainty, but their corrections were applicable only to sulfide compounds. Thus, no correction scheme applicable to broad chemical classes and structures has included full uncertainty quantification for the fitted energy corrections.

Accordingly, in this work we present a technique to quantify the uncertainty in DFT energy corrections in a way that accounts for both experimental uncertainty and the selection of fit parameters. We incorporate this technique into a new DFT energy correction scheme comprising a mixture of oxidation-state and composition-dependent corrections. After computing the corrections, we then examine how the associated uncertainties affect predicted formation energies and phase stability.

## Results and Discussion

### Correction Scheme

Our correction scheme combines aspects of several previous schemes. Following Wang et al. and Jain et al.^12,14^, we compute corrections only for transition metals and anion species expected to exhibit systematic errors (see Table 1), and employ a mixture of GGA and GGA+*U* calculations for selected elements, as explained below. However, whereas previous schemes fit corrections for each specie separately, we fit all corrections simultaneously using a system of linear equations (similar to the FERE method), where uncertainties are obtained as the standard deviations from the fitting procedure. Compared to fitting species individually, this approach allows us to include more compounds (e.g., ternaries containing multiple corrected species) and to capture cross-correlation effects between species.

**Table 1:** Fitted energy corrections, uncertainties, and types of compounds to which each correction is applied.

Species	Correction (eV/atom)	Uncertainty (eV/atom)	Applies to
Oxide	− 0.687	0.0020	oxides
Peroxide	− 0.465	0.0172	peroxides
Superoxide	− 0.161	0.0075	superoxides
S	− 0.503	0.0093	anion cpds.
H	− 0.179	0.0013	anion cpds.
F	− 0.462	0.0026	anion cpds.
Cl	− 0.614	0.0018	anion cpds.
Br	− 0.534	0.0026	anion cpds.
I	− 0.379	0.0055	anion cpds.
N	− 0.361	0.0093	anion cpds.
Se	− 0.472	0.0341	anion cpds.
Si	0.071	0.0165	anion cpds.
Sb	− 0.192	0.0089	anion cpds.
Te	− 0.422	0.0262	anion cpds.
V	− 1.700	0.0064	TM oxides and fluorides
Cr	− 1.999	0.0108	TM oxides and fluorides
Mn	− 1.668	0.0053	TM oxides and fluorides
Fe	− 2.256	0.0101	TM oxides and fluorides
Co	− 1.638	0.0060	TM oxides and fluorides
Ni	− 2.541	0.0107	TM oxides and fluorides
W	− 4.438	0.0253	TM oxides and fluorides
Mo	− 3.202	0.0089	TM oxides and fluorides

These are the correction values used in the Materials Project database at the time of publication (database version 2021.05.13). The corrections may be periodically refit in the future using the method described herein.

In contrast to the FERE method^21,22^ which applies corrections to all compounds, our scheme applies corrections only to three specific categories of compounds. First, corrections for O species labeled with words—‘oxide’, ‘superoxide’, and ‘peroxide’—are applied only to compounds containing O in a specific bonding environment, as determined from nearest-neighbor bond lengths (e.g., <1.35 Å for ‘superoxide’, <1.49 Å for ‘peroxide’, and ‘oxide’ otherwise). Thus, Na$_2$O receives an ‘oxide’ correction while NaO$_2$ receives a ‘superoxide’ correction. Second, specie corrections labeled with element symbols (e.g., ‘N’, ‘H’, or ‘Si’) are applied to any compound containing that element as an anion. For example, the ‘H’ correction is applied to LiH but not to H$_2$O. A specie is classified as an anion if its estimated oxidation state (when available) is negative, or if it is the most electronegative element in the formula. Third, transition metal specie corrections are applied only to oxide and fluoride compounds (which are calculated in GGA+*U*) and not to elemental transition metals or other compounds. The purpose of these cation corrections is to remove the error associated with mixing GGA and GGA+*U* energies, as explained by Jain et al.^14^. Finally, our correction scheme assumes independent, linear corrections associated with each specie to which a correction is fit. Thus, VO$_2$ would receive both a ‘V’ and an ‘oxide’ correction, while elemental V would receive no corrections. We note that in principle, the method we present here could be applied to fit corrections for any chemical system (as is done in the FERE method^21,22^). However, we chose to correct only the above three categories of compounds in an attempt to address the most systematic and significant sources of DFT error while minimizing the amount of empiricism in our phase stability calculations.

We fit energy corrections using a set of 222 binary and ternary compounds from the Materials Project database^26^ for which both DFT and experimental enthalpies of formation were available (see Methods section and Supplementary Table S1). The DFT energies of these compounds comprise both GGA and GGA+*U* calculations, with *U* values fit according to Jain et al.^14^ (see Supplementary Table S3) and applied only to transition metal compounds that contain oxygen or fluorine. We approximate the DFT formation enthalpy at room temperature of these compounds, $\Delta H_f^{o,298K,expt}$, as the formation enthalpy calculated from DFT energies at 0 K. Further details of the compound set, fitting procedure, and correction scheme are provided in Methods section.

### Corrections and Uncertainties

Fitted energy corrections and associated uncertainties are shown in Table 1, along with a brief description of which compound category each correction is applied to. With the exception of Si, the corrections are all negative, indicating that uncorrected GGA and GGA+*U* energies generally underpredict the magnitude of the formation enthalpy, $\Delta H_f$. This underprediction is attributable to GGA’s overbinding of diatomic gas molecules^11^ or (in the case of the transition metal cations) to errors associated with mixing GGA and GGA+*U* energies^14^.

In most cases where previous studies^11,12,14^ fit energy corrections to the same species we fit in this study, our corrections were within approximately 0.1 eV/atom of those reported in literature (see Supplementary Table S2); however, the Fe, Ni, and Mo corrections differed by a larger amount (approximately 0.5 eV/atom). The fact that we sometimes obtain substantially different correction values compared to previous studies reflects the fact that (i) we fit to a larger, more diverse data set, (ii) we fit all compounds simultaneously, and (iii) some studies may have used DFT energies from other databases that employ different calculation settings. For example, in Jain et al.^14^ the Co correction is obtained by fitting to 2 compounds, while in this work there are 8 Co compounds in the fitting data for Co.

The magnitudes of the uncertainties arising from fitting the energy corrections (2–25 meV/atom) are 1–3 orders of magnitude smaller than those of the corresponding energy corrections, and are influenced by both the compounds used in the fit and the magnitude of the associated experimental uncertainties. The uncertainties are computed as the standard deviations of the respective energy corrections, obtained by minimizing the sum of squared errors, weighted by experimental uncertainty (see Correction Fitting Procedure section). As such, they reflect both uncertainty in the experimental data and in the fitting procedure itself. For example, as shown in Table 1, the Se correction exhibits a large fit uncertainty, which can be understood by examining the errors in formation energy, $\delta E$, for the fitted Se compounds (Supplementary Figure S1). One of the five compounds, CaSe, exhibits a large experimental uncertainty and does not conform to the linear model our scheme assumes (i.e., its $\delta E$ is substantially different from that of other compounds with similar Se content). Because individual compounds are weighted by their experimental uncertainty when fitting energy corrections (see Methods section), this compound does not significantly affect the value of the fitted correction. However because it also does not conform well to the linear model we assume, it does substantially increase the associated fit uncertainty.

Fitting all corrections simultaneously also enables us to extract information about cross-correlation effects (i.e., DFT errors associated with the co-occurrence of two species). For example, the obtained cross-correlation or covariance matrix (Supplementary Figure S2) shows that there is a large covariance between the Mo and O corrections (i.e., the covariance is similar in magnitude to the average uncertainty in the respective corrections). This result indicates that these two species influence one another’s DFT formation energies, partially violating the assumption of independent, linear corrections.

### Corrected Formation Energies

**Figure 1:**
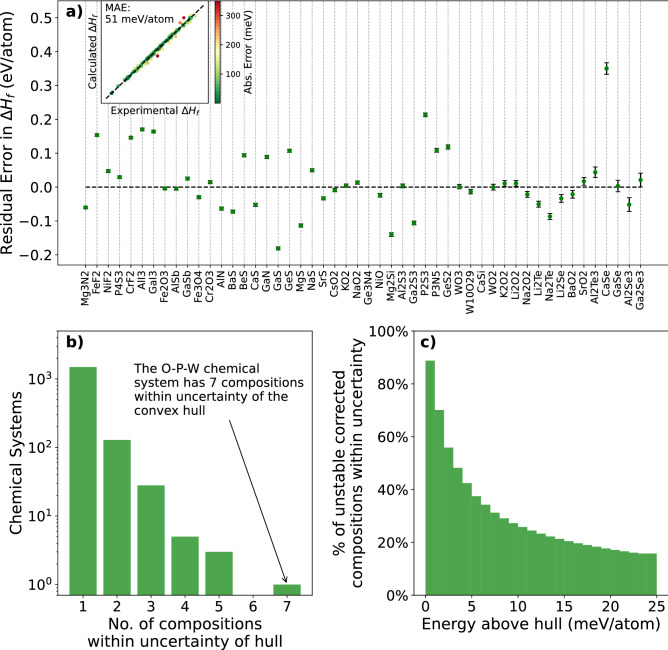
Fitted energy corrections and uncertainties. (**a**) Residual error in calculated formation energy ($\Delta H_f^{o,298K,expt} - \Delta H_f^{o,298K,DFT}$) for the 50 compounds with the largest fit uncertainty, which is indicated by the error bars. Compounds are sorted by increasing fit uncertainty such that the highest fit uncertainties are listed on the right hand side of the plot. Inset: calculated and experimental formation energies for the entire dataset after fitting the corrections. The dashed line indicates perfect agreement. (**b**) Number of chemical systems in the Materials Project database^26^ that contain unstable phases within fit uncertainty of the ground-state energy hull. (**c**) Percent of unstable compositions that contain corrections and are within uncertainty of the hull at a given energy above hull.

The fitted corrections enabled accurate prediction of formation energies, with an MAE of 51 meV/atom and a root mean squared error (RMSE) of 92 meV/atom across the entire dataset of 222 compounds (see Fig. 1a inset). In this respect, our method achieved comparable accuracy to previously-reported correction schemes^14,15,22^ which reduced the error in GGA or GGA+*U* formation enthalpies from $\approx$ 175–450 meV/atom uncorrected to $\approx$ 45–55 meV/atom after correction. In addition to accuracy, our scheme enables calculation of the propagated uncertainty in the formation energies that arises from the energy corrections applied to each compound. Figure 1a shows how the calculated uncertainty in formation energy compares to the residual error for the compounds with the largest fit uncertainty. While in most cases the uncertainty is smaller in magnitude than the residual error, for a few compounds (for example CaSi, near the far right) the uncertainty is similar to the residual error.

To be rigorous, it should be noted that the calculated formation energy uncertainties are not necessarily statistically independent for compounds within the same chemical system. Uncertainties are computed on a specie-by-specie basis, and hence the formation energies of two or more compounds may be shifted in the same direction by the corrections. This is particularly true for polymorphs which share the same composition. As a result, the relative differences between calculated energies of different compounds in the same chemical system would be similar regardless of the error in the correction value. Since the degree of statistical dependence is unknown, and since accounting for it would only decrease the associated uncertainty, we treat all formation energy uncertainties of (non-polymorphic) compounds as statistically independent here. It is also important to note that the uncertainties we compute here capture only uncertainties arising from the fitting procedure and experimental data, not in the functional itself. Hence, the uncertainties we compute here may underestimate the total error in the corrected DFT formation energy. We expect our assumption of statistical independence, which increases the computed uncertainty, to partially cancel this underestimation.

### Implications for Phase Diagram Construction

The fit uncertainty in the corrected DFT formation energies can be as large as several meV/atom, as shown in Fig. 1a, which is comparable to the energy above the hull for unstable polymorphs in many chemical systems. Quantifying this uncertainty can reveal cases in which the ability of empirically-corrected DFT calculations to differentiate between stable and unstable phases may be limited. Figure 1b shows that there are many chemical systems which have one or more unstable compositions within uncertainty of the energy convex hull^27^. Among unstable compositions that have corrections, roughly 50% of those within 5 meV of the hull are within uncertainty (Fig. 1c).

Since the estimated stability of a particular phase in a compositional phase diagram is impacted by not only the energy of that phase, but also that of neighboring phases, even relatively small uncertainties can have significant effects on predicted phase stability. To illustrate this, we used a bootstrapping approach to determine the approximate distribution of possible compositional phase diagrams, considering uncertainties, for the Sc–W–O chemical system. The only known ternary compound in this system is scandium tungstate, Sc$_2$(WO$_4$)$_3$, which is a material studied for its unique property of negative thermal expansion^28^, as well as its open framework structure supposedly enabling trivalent ion conduction^29,30^ or even polyanion conduction^31^. The binary tungsten oxides and related substoichiometric Magnéli phases^32,33^ are also used in numerous technologies including photocatalytic^34^, photothermal^35^, and electrochromic^36^ applications. W has the highest uncertainty (0.0253 eV/atom) among the transition metals in our correction scheme (Table 1) and the Sc–W–O system contains many competitive phases close in energy. For example, the WO$_3$ composition alone contains 25 phases within the uncertainty range of the hull (6.5 meV/atom). The formation energy of the WO$_3$ phase also impacts the reported stability of Sc$_2$(WO$_4$)$_3$, which exists directly on the Sc$_2$O$_3$–WO$_3$ facet.

**Figure 2:**
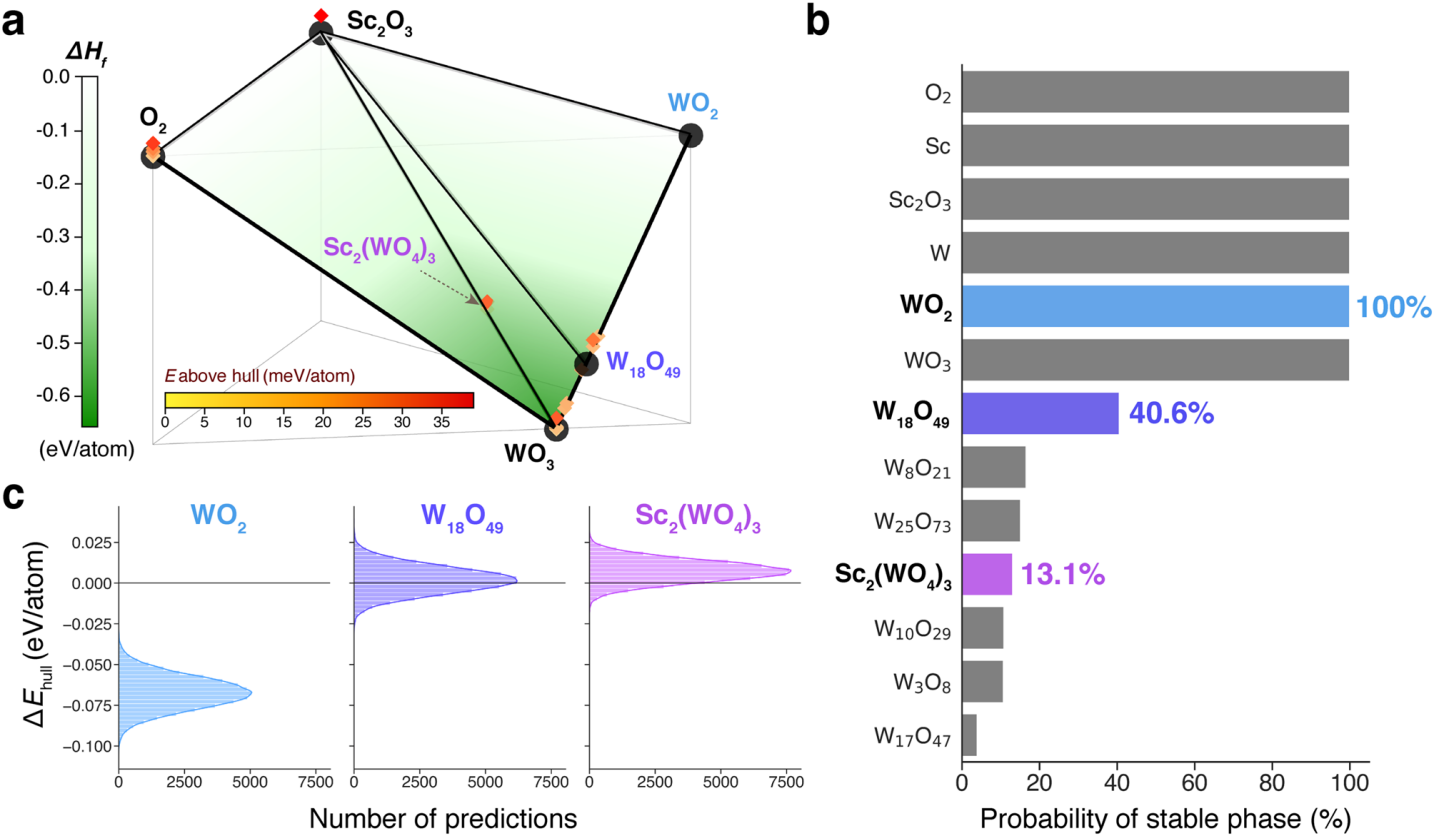
Probabilistic estimation of phase stability enabled by computed DFT energy uncertainties. (**a**) Side-view of compositional ternary phase diagram for the Sc$_2$O$_3$–WO$_2$–O$_2$ system indicating phases predicted to be stable (black circles) by DFT without considering uncertainty. Formation enthalpy, $\Delta H_f$, is denoted by the vertical axis and green shading. Unstable entries, i.e., those with a positive energy above hull, are marked as diamonds and accordingly shaded by energy above hull. (**b**) Probability that each composition in the phase diagram is stable (on the hull) as computed by a bootstrapping method that randomly samples the corrected DFT energy distributions. (**c**) Distribution of the energy to hull ($\Delta E_{\mathrm{hull}}$) for three selected compositions after 50,000 simulations. WO$_2$ and W$_{18}$O$_{49}$ are predicted to be stable by DFT without considering uncertainty. Sc$_2$(WO$_4$)$_3$ is predicted to be narrowly unstable by previous DFT calculations, but is known to be experimentally stable.

The baseline compositional phase diagram, without considering uncertainties, is shown in Fig. 2a. One substoichiometric tungsten oxide composition, (W$_{18}$O$_{49}$), is predicted to be stable by DFT without considering uncertainties and the Sc$_2$(WO$_4$)$_3$ composition is predicted to be slightly unstable (above the hull) despite experimental evidence indicating its stability^37^. Many other substoichiometric tungsten oxide phases are very close to the hull.

Phase stability probabilities were assessed by constructing 50,000 phase diagrams with DFT energies randomly sampled from their predicted energy range with uncertainty (see Methods). Figure 2b shows the probability that a particular phase was stable (on the hull) in the set of constructed phase diagrams. Figure 2c shows the distribution of energy “to” hull, $\Delta E_{\mathrm{hull}}$, for the three selected compositions, where a positive $\Delta E_{\mathrm{hull}}$ corresponds to the conventional energy above hull and a negative $\Delta E_{\mathrm{hull}}$ indicates the decomposition enthalpy to the stable phase from the set of nearest stable phases (i.e., energy *below* hull). The calculated $\Delta E_{\mathrm{hull}}$ values were − 0.067 ± 0.010, 0.002 ± 0.008, and 0.007 ± 0.007 eV/atom for WO$_2$, W$_{18}$O$_{49}$, and Sc$_2$(WO$_4$)$_3$ respectively. These values differ from the values that would be predicted when using correction uncertainties independently, due to the dependence of a phase’s stability on the energies of its neighbors and the probabilistic nature of these calculations. In fact, the standard deviations of the $\Delta E_{\mathrm{hull}}$ distributions are slightly larger ($\sim$ 1.1 to 1.4 times) than the phase’s corresponding correction uncertainty, indicating that phase stability predictions are even more sensitive to uncertainties due to the simultaneous consideration of multiple uncertainties.

The probabilistic phase stability analysis for the Sc–W–O system illustrates how the quantification of the uncertainty in corrected DFT formation energies enables better-informed assessment of the relative (meta)stability of different phases. These results suggest that some phases that are experimentally stable, but reported metastable by DFT, may *actually* be stable in DFT within the uncertainty of applied corrections. Furthermore, some existing phases which are predicted to be stable but are very close in energy to their neighbors (e.g., W$_{18}$O$_{49}$) have a significant chance ($\sim$ 60%) of being reported as metastable. Thus even without considering uncertainties in the DFT calculations themselves, the quantification of uncertainties in the energy corrections is significant enough to impact the results of phase stability predictions. Hence we recommend the careful consideration of correction uncertainties, particularly for challenging chemical systems, in phase stability analyses using DFT-computed energies.

### Outlook

In summary, we have described a methodology to quantify uncertainty in fitted DFT energy corrections that arises from both the underlying experimental data and the fitting procedure. We demonstrated this method in a correction scheme comprising a mixture of oxidation-state and composition-dependent corrections that achieved an MAE of 51 meV/atom over a set of 222 binary and ternary compounds. We showed that uncertainties in corrected DFT energies are large enough to encompass multiple unstable compositions in many chemical systems, and that such uncertainties can be used to estimate the probability of phase stability.

Towards continued progress in the area of empirical DFT corrections, we note several limitations of the correction scheme we presented here. First, the assumption of independent, linear energy corrections applied to each species clearly breaks down in some compounds, such as those containing polyanions. The ability of our scheme to calculate cross-correlation effects between species is helpful for identifying chemistries that violate this linear assumption. Second, we note that our scheme has only a limited ability to distinguish between different bonding environments for the same element (e.g., oxide vs. superoxide). Fitted corrections for other species may fail to accurately reproduce the error of compounds containing that specie in a different oxidation state. For example, our fitted corrections for N do a poor job of describing the formation enthalpy of azides. We view correcting oxide formation energies as especially critical due to the predominance of oxides in nature and in materials science research (hence our differentiation between oxide, peroxide, and superoxide compounds). For other applications (e.g., in chemistry or physics) in which compounds containing anions other than oxygen may predominate, a more sophisticated correction scheme may be warranted. For example, the “Coordination-Corrected Formation Enthalpy” (CCE) method of Friedrich et al.^16^ treats polyanions and elements with multiple oxidation states more accurately than the scheme presented here, albeit at the expense of a more complicated set of fitting requirements. Third, we also note that our scheme and more sophisticated correction schemes may be practically limited by the amount and quality of experimental formation energy data available for ternary compounds. Our scheme generally benefits from adding more ternary data because this both reduces the uncertainty in the fitted corrections and increases the amount of information available for calculating cross-correlation effects between species. Moreover, the methods used to compute uncertainties in experimental formation energies may vary among data sources, making it difficult to assign a rigorous interpretation to the meaning of our fitted uncertainties. Although a method to precisely and fully quantify all sources of uncertainty in a DFT calculation remains elusive, we believe our framework provides a useful step in this direction that can enhance the quality of phase stability predictions.

## Methods

### Compound Selection

Building on the correction schemes of Wang et al. and Jain et al.^12,14^, we fit energy corrections to 14 anion species—oxide, peroxide, superoxide, S, F, Cl, Br, I, N, H, Se, Si, Sb, Te—and 8 transition metal cations—V, Cr, Mn, Fe, Co, Ni, W, and Mo. We calculate transition metal oxide and fluoride compound energies using GGA+*U*, and all other energies in GGA. Jain et al.^14^ showed that the error in reaction energies that is introduced by mixing GGA and GGA+*U* calculations can be removed by a constant energy correction applied to each transition metal in the GGA+*U* compound. This is the function of the transition metal energy corrections in our scheme, while the remaining species in our list are anions that display systematic errors that do not completely cancel when calculating formation energies from the corresponding pure elements^11,12^.

Because we use GGA+*U* only for transition metal oxide and fluoride compounds, all the transition metal compounds in the fitting set contain either oxygen or fluorine, and the resulting cation energy corrections are applied only to oxide and fluoride compounds. Elemental transition metals or other transition metal compounds do not receive an energy correction. Corrections for O species labeled with words—‘oxide’, ‘superoxide’, and ‘peroxide’—are fit only to compounds containing oxygen in a specific bonding environment, as determined by the oxide_type algorithm in pymatgen^38^, which classifies *O–O* bonds as ‘superoxide” if shorter than 1.35 Å, ‘peroxide” if shorter than 1.49 Å, and ‘oxide’ otherwise. Corrections labeled with element symbols (e.g., ‘N’, ‘H’, or ‘Si’) are fit to any compound containing that element as an anion. A specie is classified as an anion if its estimated oxidation state (generated using the oxi_state_guesses method or BVAnalyzer class in pymatgen^38^, for example) is negative. If estimated oxidation states are not available, then the specie is considered an anion if it is the most electronegative element in the formula. For example, the ‘H’ anion correction is applied to LiH but not to H$_2$O.

Our initial set of fitting compounds comprised any binary or ternary compound for which both DFT energies and experimental energies were available that contained either (1) a corrected transition metal and oxygen or fluorine or (2) one or more corrected anion species and at least one main group element. Compounds containing lanthanoid, actinoid, or post-transition metal elements (except for Ga and Al) were excluded, as were compounds containing B and As or any of the polyanions –SO$_3$, –SO$_4$, –CO$_3$, –OCl$_3$, –ClO$_3$, –ClO$_4$, –NO$_2$, –NO$_3$, –PO$_3$, –PO$_4$, –OH, –P$_2$O$_7$, –SiO$_3$, –SiO$_4$, –Si$_2$O$_5$, –SeO$_3$, –TiO$_3$, –TiO$_4$, or WO$_4$. All these compounds were excluded because they consistently appeared as severe outliers when fitting corrections, indicating that they do not conform well to the model of independent, linear corrections that our scheme assumes. We also excluded the compound WOF$_4$ and any compounds that exhibited large experimental uncertainties (>10% relative uncertainty) or were predicted to be unstable by DFT (energy above hull >100 meV/atom). The resulting set of fitting data comprises 222 compounds, listed in Supplementary Table S1, of which 156 have quantified uncertainties. Each of these materials was matched to a computed structure in the Materials Project database as described in the Computed and Experimental Data Sources section below.

### Correction Fitting Procedure

For each compound, we take the difference between the experimental and calculated formation enthalpies at 1 atm and 298 K ($\delta E = \Delta H_f^{o,298K,expt} - \Delta H_f^{o,298K,DFT}$) and equate it to a linear combination of individual specie energy corrections weighted by the stoichiometric coefficients of the corresponding species. For example, to compute the energy corrections for Li$_2$O, KF, and V$_2$O$_5$, we would create the following system of linear equations:

$$\begin{aligned}\delta E(Li_2O) = \epsilon _{oxide}\\\delta E(KF) = \epsilon _{F}\\\delta E(V_2O_5) = 5\epsilon _{oxide} + 2\epsilon _{V}\end{aligned}$$

where $\epsilon _{specie}$ is the energy correction for that specie. We compute the corrections simultaneously by solving the linear system $A\epsilon = \delta E$, where *A* is a matrix of stoichiometric coefficients, $\epsilon$ is a vector of corrections, and $\delta E$ a vector of the energy differences. The resulting equation for our example system would be:

$$\begin{aligned} \begin{bmatrix} 1 &{} 0 &{} 0 &{} 0 &{}\dots \\ 0 &{} 0 &{} 0 &{} 1 &{} \dots \\ 5 &{} 0 &{} 0 &{} 0 &{} \dots &{} 2 &{} \dots \\ \end{bmatrix} \begin{bmatrix} \epsilon _{oxide}\\ \epsilon _{peroxide}\\ \epsilon _{superoxide}\\ \epsilon _{F}\\ \vdots \\ \epsilon _{V}\\ \vdots \end{bmatrix} = \begin{bmatrix} \delta E_{Li_2O}\\ \delta E_{KF}\\ \delta E_{V_2O_5}\\ \end{bmatrix} \end{aligned}$$

where $\epsilon$ is the energy correction. To solve the system, we use linear regression to obtain the specie corrections, $\epsilon _i$, that minimize the sum of the squared residuals, weighted by the experimental uncertainties:

1 $$\begin{aligned} \sum _i (\frac{\delta E_{i, predicted} - \delta E_{i, actual}}{\sigma _{H^{o}_i}})^2 \end{aligned}$$

where $\sigma _{H^{o}_i}$ is the experimental uncertainty in $\Delta H_f^{o,298K,expt}$ for compound *i* and $\delta E_{i, predicted}=\sum _i A_i \epsilon _i$. Thus, compounds with high experimental uncertainties exert a smaller influence on the fitted $\epsilon _i$. For compounds where the experimental uncertainty was not available, we assigned $\sigma _{H^{o}_i}$ a value equal to the average uncertainty of all other compounds. This was done to prevent compounds with unknown experimental uncertainty from having a disproportionately large weight when fitting corrections. We report the uncertainty of each fitted correction as the standard deviation of $\epsilon _i$ obtained from the linear regression^39^.

Following previous works^12,14,16^, we approximate the standard formation enthalpy at 298 K and atmospheric pressure as the change in DFT energy at 0 K and 0 atm:

$$\begin{aligned}\Delta H_{f_{A_{n_A}B_{n_B}C_{n_C}}^{o,298K,DFT}} \approx E^{0K,DFT}_{A_{n_A}B_{n_B}C_{n_C}} - \sum _{i=A,B,C} n_i E^{0K,DFT}_{i}\end{aligned}$$

where *H* is the enthalpy, *E* is the DFT energy and *A*, *B*, and *C* represent constituent elements. This formulation neglects changes in internal energy between 0 K and 298 K due to pressure-volume effects, vibrational effects, and zero point energy. In solids, the zero-point energy and pressure-volume effects are small (on the order of a few meV/atom^16,18^), while vibrational effects can cause $H^{298K}$ and $H^{0K}$ to differ by up to 20–30 meV/atom^16,21^. However, it has been shown that the dependence of *H* on temperature is remarkably similar among crystalline solids^40^, and hence differences between $H^{298K}$ and $H^{0K}$ will largely cancel out when computing solid–solid energy differences, as we do here. Considering that any uncancelled finite-temperature error will be much smaller in magnitude than the formation energies we are fitting (hundreds to thousands of meV/atom) and that our ultimate objective is to predict $\Delta H_f^{o,298K,expt}$ from DFT energies, we absorb this error into our fitted corrections rather than try to explicitly estimate $\Delta H_f^{o,0K,expt}$.

### Probabilistic Phase Stability Analysis

Phase stability distributions were approximated using a bootstrapping technique, in which the energy of each phase was randomly sampled from a normal distribution with standard deviation equal to the composition’s DFT uncertainty before performing convex hull analysis in energy-composition space to yield the compositional phase diagram. We calculated 50,000 compositional phase diagrams for the Sc–W–O example chemical system and examined the resulting energy to hull ($\Delta E_{\mathrm{hull}}$) distributions for each phase. A compound was determined to be stable if $\Delta E_{\mathrm{hull}} \le 0$, and unstable otherwise. During phase diagram construction, we considered only the lowest energy phases for each composition; i.e., non-ground state polymorphs were excluded. This decision was made to account for the lack of statistical independence in energies for phases which share the same composition. Hence for the purpose of stability analysis, we assumed the relative ordering of polymorphs for a given composition did not change during the random sampling of the hull.

### Computed and Experimental Data Sources

DFT energies for the compounds were obtained from the Materials Project database^26^ version 2021.03.22 and comprise a combination of GGA and GGA+*U* calculations, with *U* values fit according to the procedure of Jain et al.^14^ (see Supplementary Table S3). Note that *U* values were fit to minimize the error in thermochemical properties rather than, e.g., band gaps or lattice parameters. For magnetic materials, the ground-state magnetic orderings were identified using an automated workflow^41^ and are listed in Supplementary Table 1.

Experimental formation enthalpy data were compiled primarily from the Kubaschewski tables^42^, the NIST JANAF database^43^, and the compilation of Kim et al.^44,45^. Data from the Landolt Bornstein database^46^ was included for FeMoO$_4$, Na$_4$V$_2$O$_7$, Na$_2$MoO$_4$, KFeO$_2$ because this data is considered more reliable than older values^18^. Formation enthalpies for Ag$_2$O, Ag$_2$O$_3$ and AgO were taken from the CRC Handbook^47^, and the value for AlFe was from Ryzman et al.^48^. Values for binary hydrides, oxides, nitrides, chlorides, and fluorides compiled in Grindy et al.^11^ were also included. After aggregating the data, we removed data for pure elements and compounds that are liquids or gases at 298 K, leaving a total of 2,600 formation enthalpy values.

Next, we deduplicated the data to identify a single formation enthalpy value for each compound. For many compounds, values from newer datasets (e.g., the NIST JANAF database) were identical to or within 1 meV/atom of values in the Kubaschewski tables, except that the newer values lacked uncertainties. In such cases, we retained the Kubaschewski value, with uncertainty, and discarded the newer values. Formulas for which there was a large disagreement among databases (e.g. greater than 200 meV/atom) were manually inspected to identify and correct any typographical or scaling errors. After this procedure, the dataset contained 192 compounds with exactly 2 experimental values (differing by more than 1 meV/atom), 34 compounds with 3 values, and 3 compounds (Al$_2$SiO$_5$, B$_2$O$_3$, and SiO$_2$) with 4 values. For compounds with 2 experimental values, if one value was from the Kubaschewski tables and the other values was from a newer source, we retained the newer value and discarded the Kubaschewski value. If neither value was from the Kubaschewski tables, we retained the value with the lowest uncertainty. For compounds with three values, we retained the value closest to the average. In the case of Al$_2$SiO$_5$, the multiple values were associated with different polymorphs. Here, we retained the polymorph with the lowest formation energy (kyanite). For B$_2$O$_3$, we retained the value for the hexagonal phase from Kubaschewski, and for SiO$_2$ we retained the $\alpha$-crystobalite phase from Kubaschewski.

Finally, we matched compounds in the formation energy dataset to likely Materials Project IDs (mpids). First, we compiled candidate mpids by querying the Materials Project API^49^ (database version 2021.03.22) for materials that were (1) not marked ‘theoretical’, (2) matched at least one ICSD^50,51^ material, and (3) were within 200 meV/atom of the DFT-computed energy hull. Among these candidates, we chose the material with the lowest e_above_hull whose spacegroup matched the spacegroup reported in the original data (when available). If no spacegroup was reported, we chose the candidate with the lowest e_above_hull. Finally, we manually validated (and adjusted where necessary) the mpids of Al$_2$SiO$_5$ (kyanite) as well as the magnetic compounds Fe$_3$O$_4$, MnO, Co$_3$O$_4$, CoO, NiO, CuO, VO$_2$, MnO$_2$, and Mn$_2$O$_3$, whose experimental ground states are likely to be mispredicted by DFT. For the magnetic compounds, we assigned mpids to match the ground state spacegroups listed in Table I of Wang et al.^12^. The resulting mpids are labeled likely_mpid in the dataset to signify that most were not individually validated. However, likely_mpid were checked against mpids assigned by the compilers of the original data in Kim et al.^45^, and any discrepancies were manually investigated and corrected when appropriate.

In total, there are 2,135 unique materials in the formation enthalpy dataset, of which 1,834 include uncertainties and 1,580 have an associated likely_mpid. The dataset is provided as Supporting Information with this manuscript and will also be integrated into Matminer^52^ in the near future.

## Data Availability

The correction scheme described in this work has been integrated into pymatgen^38^ as of release 2022.0.8 and is the scheme currently used in the Materials Project database^26^ beginning with version 2021.05.13. A new CorrectionCalculator class is provided within pymatgen for readers interested in reproducing our results or applying our scheme to their own data. Given a set of calculations, experimental data, and a list of species whose energies are to be corrected, the CorrectionCalculator class automatically fits the energy corrections and uncertainties and generates an input file that can be used to apply the corrections within pymatgen. A Jupyter notebook demonstrating the use of this new class as well as archive files containing the DFT energies and experimental data we used are provided as Supplementary Information.

## Supplementary Information

Supplementary Information 1.

Supplementary Information 2.
