# Supplementary material for: A framework for quantifying uncertainty in DFT energy corrections
Source: Sci Rep. 2021 Jul 29;11:15496. doi: 10.1038/s41598-021-94550-5 (PMC8322326; doi:10.1038/s41598-021-94550-5)
Supplement: Supplementary file 1 — Supplementary Information 1. [file 41598_2021_94550_MOESM1_ESM.pdf]

A framework for quantifying uncertainty in DFT energy corrections

Supplementary Information

Amanda Wang[1,*], Ryan Kingsbury[1,*], Matthew McDermott[1,2], Matthew Horton[1,2], Anubhav Jain[2], Shyue Ping Ong[2,3], Shyam Dwaraknath[2], and Kristin A. Persson[1,2,4,+]

[1]Department of Materials Science and Engineering, University of California, Berkeley, CA 94720

[2]Materials Sciences Division, Lawrence Berkeley National Laboratory, 1 Cyclotron Road, Berkeley, CA 94720

[3]University of California San Diego, La Jolla, CA 92093

[4]Molecular Foundry, Lawrence Berkeley National Laboratory, 1 Cyclotron Road, Berkeley, CA 94720

[*]These two authors contributed equally

[+]Correspondence to kapersson@lbl.gov

Table S1: Experimental and calculated formation energies and uncertainties (eV/atom) for the compounds used to fit corrections. See the main text for details of how the data were obtained.

Compound	Experimental Formation Energy	Experimental Uncertainty	Corrected DFT Formation Energy	Corrected DFT Uncertainty	Magnetic Ordering
Al_2FeO_4	-2.91	$3.7 \cdot 10^{-3}$	-2.9	$1.84 \cdot 10^{-3}$	AFM
Al_2NiO_4	-2.84	$6.81 \cdot 10^{-3}$	-2.78	$1.91 \cdot 10^{-3}$	AFM
Al_2O_3	-3.46	–	-3.43	$1.2 \cdot 10^{-3}$	–
Al_2S_3	-1.35	–	-1.35	$5.58 \cdot 10^{-3}$	–
Al_2Se_3	-1.18	$5.2 \cdot 10^{-2}$	-1.12	$2.05 \cdot 10^{-2}$	–
Al_2SiO_5	-3.35	$2.72 \cdot 10^{-3}$	-3.36	$1.25 \cdot 10^{-3}$	–
Al_2Te_3	-0.66	$1.91 \cdot 10^{-2}$	-0.7	$1.57 \cdot 10^{-2}$	–
$AlBr_3$	-1.32	–	-1.52	$1.95 \cdot 10^{-3}$	–
$AlCl_3$	-1.83	$4.4 \cdot 10^{-3}$	-1.97	$1.35 \cdot 10^{-3}$	–
AlF_3	-3.91	$3.37 \cdot 10^{-3}$	-3.87	$1.95 \cdot 10^{-3}$	–
AlH_3	$-2.95 \cdot 10^{-2}$	$2.07 \cdot 10^{-3}$	-0.14	$9.75 \cdot 10^{-4}$	–
AlI_3	-0.78	–	-0.96	$4.12 \cdot 10^{-3}$	–
AlN	-1.65	–	-1.58	$4.65 \cdot 10^{-3}$	–
AlSb	-0.26	$5.18 \cdot 10^{-3}$	-0.26	$4.45 \cdot 10^{-3}$	–
$BaAl_2O_4$	-3.44	$1.17 \cdot 10^{-2}$	-3.45	$1.14 \cdot 10^{-3}$	–
$BaBr_2$	-2.62	$5.87 \cdot 10^{-3}$	-2.58	$1.73 \cdot 10^{-3}$	–
$BaCl_2$	-2.97	–	-2.97	$1.2 \cdot 10^{-3}$	–
BaF_2	-4.18	–	-4.16	$1.73 \cdot 10^{-3}$	–
BaH_2	-0.65	–	-0.61	$8.67 \cdot 10^{-4}$	–
BaI_2	-2.09	–	-2.05	$3.67 \cdot 10^{-3}$	–
$BaMoO_4$	-2.62	$1.16 \cdot 10^{-2}$	-2.65	$1.99 \cdot 10^{-3}$	–
BaO	-2.84	$1.09 \cdot 10^{-2}$	-2.82	$1 \cdot 10^{-3}$	–
BaO_2	-2.19	$4.04 \cdot 10^{-2}$	-2.17	$1.15 \cdot 10^{-2}$	–
BaS	-2.4	$1.71 \cdot 10^{-2}$	-2.33	$4.65 \cdot 10^{-3}$	–
BaV_2O_6	-2.63	$7.72 \cdot 10^{-3}$	-2.69	$1.95 \cdot 10^{-3}$	–
Be_3N_2	-1.22	–	-1.22	$3.72 \cdot 10^{-3}$	–
$BeAl_2O_4$	-3.41	$8.74 \cdot 10^{-3}$	-3.35	$1.14 \cdot 10^{-3}$	–
$BeAl_6O_{10}$	-3.43	$6.1 \cdot 10^{-3}$	-3.4	$1.18 \cdot 10^{-3}$	–
$BeBr_2$	-1.23	$4.35 \cdot 10^{-2}$	-1.4	$1.73 \cdot 10^{-3}$	–
$BeCl_2$	-1.71	$1.14 \cdot 10^{-2}$	-1.84	$1.2 \cdot 10^{-3}$	–
BeF_2	-3.55	$1.45 \cdot 10^{-2}$	-3.55	$1.73 \cdot 10^{-3}$	–

Table S1 – continued from previous page

BeO	−3.12	–	−3.1	$1 \cdot 10^{-3}$	–
BeS	−1.21	–	−1.31	$4.65 \cdot 10^{-3}$	–
$Ca_2Fe_2O_5$	−2.46	$6.22 \cdot 10^{-3}$	−2.46	$2.5 \cdot 10^{-3}$	FiM
$Ca_2V_2O_7$	−2.9	$6.31 \cdot 10^{-3}$	−2.91	$1.72 \cdot 10^{-3}$	–
$Ca_3Al_2O_6$	−3.38	$4.33 \cdot 10^{-3}$	−3.4	$1.09 \cdot 10^{-3}$	Unknown
Ca_3N_2	−0.89	–	−0.93	$3.72 \cdot 10^{-3}$	–
$Ca_3Si_2O_7$	−3.41	$1.09 \cdot 10^{-2}$	−3.46	$1.17 \cdot 10^{-3}$	–
Ca_3SiO_5	−3.37	$9.1 \cdot 10^{-3}$	−3.42	$1.11 \cdot 10^{-3}$	–
$Ca_3V_2O_8$	−3.01	$6.7 \cdot 10^{-3}$	−2.97	$1.58 \cdot 10^{-3}$	–
Ca_3WO_6	−3.04	$1.69 \cdot 10^{-2}$	−3.06	$2.8 \cdot 10^{-3}$	–
$Ca(FeO_2)_2$	−2.19	–	−2.17	$3.1 \cdot 10^{-3}$	FM
$CaAl_2O_4$	−3.44	$3.11 \cdot 10^{-3}$	−3.45	$1.14 \cdot 10^{-3}$	–
$CaAl_4O_7$	−3.45	$2.85 \cdot 10^{-3}$	−3.46	$1.17 \cdot 10^{-3}$	–
$CaBr_2$	−2.36	–	−2.4	$1.73 \cdot 10^{-3}$	–
$CaCl_2$	−2.75	–	−2.8	$1.2 \cdot 10^{-3}$	–
$CaCr_2O_4$	−2.71	–	−2.7	$3.29 \cdot 10^{-3}$	FiM
CaF_2	−4.24	–	−4.22	$1.73 \cdot 10^{-3}$	–
$CaGeO_3$	−2.66	$1.12 \cdot 10^{-2}$	−2.75	$1.2 \cdot 10^{-3}$	–
CaH_2	−0.6	–	−0.67	$8.67 \cdot 10^{-4}$	–
CaI_2	−1.85	–	−1.89	$3.67 \cdot 10^{-3}$	–
$CaMoO_4$	−2.67	$5.7 \cdot 10^{-3}$	−2.64	$1.99 \cdot 10^{-3}$	–
CaO	−3.29	$4.15 \cdot 10^{-3}$	−3.31	$1 \cdot 10^{-3}$	–
CaS	−2.45	$1.97 \cdot 10^{-2}$	−2.4	$4.65 \cdot 10^{-3}$	–
CaSe	−1.91	0.17	−2.26	$1.7 \cdot 10^{-2}$	–
CaSi	−0.78	$4.77 \cdot 10^{-2}$	−0.5	$8.25 \cdot 10^{-3}$	–
CaV_2O_6	−2.68	$6.22 \cdot 10^{-3}$	−2.7	$1.95 \cdot 10^{-3}$	–
Co_3O_4	−1.35	$6.81 \cdot 10^{-3}$	−1.22	$2.81 \cdot 10^{-3}$	FM
CoF_2	−2.32	$3.18 \cdot 10^{-2}$	−2.35	$2.65 \cdot 10^{-3}$	AFM
CoF_3	−2.05	–	−2.11	$2.46 \cdot 10^{-3}$	FM
CoO	−1.23	$3.11 \cdot 10^{-3}$	−1.25	$3.16 \cdot 10^{-3}$	AFM
Cr_2CoO_4	−2.12	$9.92 \cdot 10^{-3}$	−2.14	$3.4 \cdot 10^{-3}$	FiM
Cr_2CuO_4	−1.92	$1.11 \cdot 10^{-2}$	−1.94	$3.29 \cdot 10^{-3}$	FiM
Cr_2FeO_4	−2.14	$7.4 \cdot 10^{-3}$	−2.18	$3.59 \cdot 10^{-3}$	FiM
Cr_2NiO_4	−2.04	$6.22 \cdot 10^{-3}$	−2.03	$3.63 \cdot 10^{-3}$	FiM
Cr_2O_3	−2.35	$1.55 \cdot 10^{-2}$	−2.37	$4.48 \cdot 10^{-3}$	AFM

CrF_2	−2.68	$4.04 \cdot 10^{-2}$	−2.82	$4 \cdot 10^{-3}$	AFM
CrF_3	−3	$2.18 \cdot 10^{-2}$	−3.04	$3.33 \cdot 10^{-3}$	AFM
CrO_3	−1.52	$2.82 \cdot 10^{-2}$	−1.51	$3.09 \cdot 10^{-3}$	–
Cs_2CrO_4	−2.12	$4.89 \cdot 10^{-3}$	−2.07	$1.92 \cdot 10^{-3}$	–
Cs_2MoO_4	−2.24	–	−2.21	$1.71 \cdot 10^{-3}$	–
Cs_2O	−1.2	–	−1.19	$6.67 \cdot 10^{-4}$	–
CsBr	−2.1	$4.15 \cdot 10^{-3}$	−2.05	$1.3 \cdot 10^{-3}$	–
CsCl	−2.29	$2.07 \cdot 10^{-3}$	−2.25	$9 \cdot 10^{-4}$	–
CsF	−2.87	–	−2.84	$1.3 \cdot 10^{-3}$	–
CsH	−0.28	$2.07 \cdot 10^{-3}$	−0.22	$6.5 \cdot 10^{-4}$	–
CsI	−1.8	$4.35 \cdot 10^{-2}$	−1.74	$2.75 \cdot 10^{-3}$	–
CsO_2	−0.99	$7.25 \cdot 10^{-3}$	−0.98	$5 \cdot 10^{-3}$	FM
Fe_2CoO_4	−1.61	$6.81 \cdot 10^{-3}$	−1.58	$3.22 \cdot 10^{-3}$	FM
Fe_2O_3	−1.71	–	−1.71	$4.21 \cdot 10^{-3}$	AFM
Fe_3O_4	−1.66	–	−1.63	$4.48 \cdot 10^{-3}$	FM
$FeCuO_2$	−1.33	$1.09 \cdot 10^{-2}$	−1.32	$2.72 \cdot 10^{-3}$	AFM
FeF_2	−2.44	–	−2.59	$3.79 \cdot 10^{-3}$	AFM
FeF_3	−2.7	–	−2.63	$3.19 \cdot 10^{-3}$	FM
$FeMoO_4$	−1.85	–	−1.88	$2.61 \cdot 10^{-3}$	FM
Ga_2O_3	−2.26	–	−2.27	$1.2 \cdot 10^{-3}$	–
Ga_2S_3	−1.07	$2.61 \cdot 10^{-2}$	−0.96	$5.58 \cdot 10^{-3}$	–
Ga_2Se_3	−0.85	$2.61 \cdot 10^{-2}$	−0.87	$2.05 \cdot 10^{-2}$	–
$GaBr_3$	−1	$6.48 \cdot 10^{-3}$	−1.24	$1.95 \cdot 10^{-3}$	–
$GaCl_3$	−1.36	$1.19 \cdot 10^{-2}$	−1.57	$1.35 \cdot 10^{-3}$	–
GaF_3	−3.04	–	−2.93	$1.95 \cdot 10^{-3}$	–
GaI_3	−0.62	$3.14 \cdot 10^{-2}$	−0.78	$4.12 \cdot 10^{-3}$	–
GaN	−0.57	$4.77 \cdot 10^{-2}$	−0.66	$4.65 \cdot 10^{-3}$	–
GaS	−1.08	$9.74 \cdot 10^{-2}$	−0.9	$4.65 \cdot 10^{-3}$	–
GaSb	−0.23	$8.81 \cdot 10^{-3}$	−0.25	$4.45 \cdot 10^{-3}$	–
GaSe	−0.82	$6.53 \cdot 10^{-2}$	−0.83	$1.7 \cdot 10^{-2}$	–
Ge_3N_4	−0.59	–	−0.25	$5.31 \cdot 10^{-3}$	–
GeO_2	−1.93	–	−2.08	$1.33 \cdot 10^{-3}$	–
GeS	−0.39	$2.38 \cdot 10^{-2}$	−0.5	$4.65 \cdot 10^{-3}$	–
GeS_2	−0.54	$4.35 \cdot 10^{-2}$	−0.66	$6.2 \cdot 10^{-3}$	–
K_2CrO_4	−2.06	$6.22 \cdot 10^{-3}$	−2.06	$1.92 \cdot 10^{-3}$	–

K_2O	−1.25	–	−1.25	$6.67 \cdot 10^{-4}$	–
K_2O_2	−1.28	–	−1.3	$8.6 \cdot 10^{-3}$	–
K_2S	−1.3	$4.35 \cdot 10^{-2}$	−1.22	$3.1 \cdot 10^{-3}$	–
$K_2Si_4O_9$	−2.98	$1.04 \cdot 10^{-2}$	−3.07	$1.2 \cdot 10^{-3}$	–
K_3AlF_6	−3.45	$1.43 \cdot 10^{-2}$	−3.43	$1.56 \cdot 10^{-3}$	–
$KAlCl_4$	−2.07	–	−2.17	$1.2 \cdot 10^{-3}$	–
KBr	−2.04	–	−2.03	$1.3 \cdot 10^{-3}$	–
KCl	−2.26	$2.07 \cdot 10^{-3}$	−2.26	$9 \cdot 10^{-4}$	–
KF	−2.95	–	−2.94	$1.3 \cdot 10^{-3}$	–
$KFeO_2$	−1.79	–	−1.74	$2.72 \cdot 10^{-3}$	FM
KH	−0.3	$1.04 \cdot 10^{-3}$	−0.28	$6.5 \cdot 10^{-4}$	–
KI	−1.7	–	−1.68	$2.75 \cdot 10^{-3}$	–
KO_2	−0.98	$8.64 \cdot 10^{-3}$	−0.99	$5 \cdot 10^{-3}$	FM
Li_2BeF_4	−3.37	–	−3.37	$1.49 \cdot 10^{-3}$	–
Li_2O	−2.07	$3.45 \cdot 10^{-3}$	−2.06	$6.67 \cdot 10^{-4}$	–
Li_2O_2	−1.64	–	−1.65	$8.6 \cdot 10^{-3}$	–
Li_2S	−1.54	$7.25 \cdot 10^{-3}$	−1.5	$3.1 \cdot 10^{-3}$	–
Li_2Se	−1.45	$2.9 \cdot 10^{-2}$	−1.42	$1.14 \cdot 10^{-2}$	–
Li_2Te	−1.23	–	−1.18	$8.73 \cdot 10^{-3}$	–
Li_3AlF_6	−3.51	–	−3.48	$1.56 \cdot 10^{-3}$	–
Li_3N	−0.43	$3.37 \cdot 10^{-3}$	−0.46	$2.33 \cdot 10^{-3}$	–
$LiAlH_4$	−0.2	–	−0.26	$8.67 \cdot 10^{-4}$	–
$LiAlO_2$	−3.08	$1.09 \cdot 10^{-2}$	−3.08	$1 \cdot 10^{-3}$	–
LiBr	−1.82	$5.18 \cdot 10^{-3}$	−1.81	$1.3 \cdot 10^{-3}$	–
LiCl	−2.12	$2.07 \cdot 10^{-3}$	−2.12	$9 \cdot 10^{-4}$	–
LiF	−3.2	–	−3.17	$1.3 \cdot 10^{-3}$	–
$LiFeO_2$	−1.99	$1.4 \cdot 10^{-2}$	−1.92	$2.72 \cdot 10^{-3}$	FM
LiH	−0.47	$2.07 \cdot 10^{-3}$	−0.5	$6.5 \cdot 10^{-4}$	–
LiI	−1.4	–	−1.42	$2.75 \cdot 10^{-3}$	–
Mg_2Si	−0.27	–	−0.13	$5.5 \cdot 10^{-3}$	–
$Mg_2V_2O_7$	−2.67	$7.91 \cdot 10^{-3}$	−2.68	$1.72 \cdot 10^{-3}$	–
Mg_3N_2	−0.96	–	−0.9	$3.72 \cdot 10^{-3}$	–
Mg_3Sb_2	−0.62	$3.03 \cdot 10^{-2}$	−0.44	$3.56 \cdot 10^{-3}$	–
$MgAl_2O_4$	−3.4	–	−3.37	$1.14 \cdot 10^{-3}$	–
$MgBr_2$	−1.81	$8.64 \cdot 10^{-3}$	−1.86	$1.73 \cdot 10^{-3}$	–

$MgCl_2$	−2.22	–	−2.27	$1.2 \cdot 10^{-3}$	–
MgF_2	−3.88	$4.49 \cdot 10^{-3}$	−3.83	$1.73 \cdot 10^{-3}$	–
$MgGeO_3$	−2.5	$1.04 \cdot 10^{-2}$	−2.56	$1.2 \cdot 10^{-3}$	–
MgH_2	−0.26	$8.64 \cdot 10^{-3}$	−0.29	$8.67 \cdot 10^{-4}$	–
MgI_2	−1.27	$2.31 \cdot 10^{-2}$	−1.31	$3.67 \cdot 10^{-3}$	–
$MgMoO_4$	−2.42	$8.64 \cdot 10^{-3}$	−2.41	$1.99 \cdot 10^{-3}$	–
MgO	−3.12	–	−3.05	$1 \cdot 10^{-3}$	–
MgS	−1.79	$2.18 \cdot 10^{-2}$	−1.68	$4.65 \cdot 10^{-3}$	–
MgV_2O_6	−2.53	$6.22 \cdot 10^{-3}$	−2.53	$1.95 \cdot 10^{-3}$	–
Mn_2O_3	−1.99	$4.35 \cdot 10^{-3}$	−2.01	$2.44 \cdot 10^{-3}$	FM
Mn_3O_4	−2.05	$2.52 \cdot 10^{-3}$	−2.05	$2.54 \cdot 10^{-3}$	FiM
$Mn(FeO_2)_2$	−1.82	$7.4 \cdot 10^{-3}$	−1.84	$3.19 \cdot 10^{-3}$	FiM
$MnAl_2O_4$	−3.11	$9.33 \cdot 10^{-3}$	−3.05	$1.37 \cdot 10^{-3}$	AFM
MnF_2	−2.93	–	−2.97	$2.47 \cdot 10^{-3}$	AFM
$MnMoO_4$	−2.06	$1.66 \cdot 10^{-2}$	−2.07	$2.18 \cdot 10^{-3}$	FM
MnO	−1.99	$6.74 \cdot 10^{-3}$	−1.98	$2.83 \cdot 10^{-3}$	AFM
MnO_2	−1.8	$4.49 \cdot 10^{-3}$	−1.78	$2.21 \cdot 10^{-3}$	AFM
Mo_4O_{11}	−1.96	–	−1.94	$2.79 \cdot 10^{-3}$	FM
Mo_8O_{23}	−1.94	–	−1.93	$2.73 \cdot 10^{-3}$	FM
MoF_5	−2.4	$7.25 \cdot 10^{-3}$	−2.64	$2.63 \cdot 10^{-3}$	FM
MoO_2	−2.03	$5.87 \cdot 10^{-3}$	−2.02	$3.25 \cdot 10^{-3}$	FM
MoO_3	−1.93	$2.07 \cdot 10^{-3}$	−1.92	$2.68 \cdot 10^{-3}$	–
Na_2CrO_4	−1.98	$1.36 \cdot 10^{-2}$	−1.99	$1.92 \cdot 10^{-3}$	–
$Na_2Mo_2O_7$	−2.22	$1.19 \cdot 10^{-2}$	−2.11	$2.06 \cdot 10^{-3}$	–
Na_2MoO_4	−2.18	–	−2.16	$1.71 \cdot 10^{-3}$	–
Na_2O	−1.43	$2.76 \cdot 10^{-3}$	−1.44	$6.67 \cdot 10^{-4}$	–
Na_2O_2	−1.33	$1.63 \cdot 10^{-2}$	−1.31	$8.6 \cdot 10^{-3}$	–
Na_2S	−1.26	$4.35 \cdot 10^{-2}$	−1.23	$3.1 \cdot 10^{-3}$	–
Na_2SiF_6	−3.35	$4.38 \cdot 10^{-3}$	−3.38	$1.73 \cdot 10^{-3}$	–
Na_2Te	−1.16	$8.67 \cdot 10^{-2}$	−1.07	$8.73 \cdot 10^{-3}$	–
Na_3AlF_6	−3.41	–	−3.39	$1.56 \cdot 10^{-3}$	–
$Na_4V_2O_7$	−2.33	–	−2.32	$1.46 \cdot 10^{-3}$	–
$Na_5Al_3F_{14}$	−3.57	–	−3.52	$1.65 \cdot 10^{-3}$	–
$NaAlCl_4$	−1.97	–	−2.07	$1.2 \cdot 10^{-3}$	–
$NaAlO_2$	−2.94	–	−2.93	$1 \cdot 10^{-3}$	–

Table S1 – continued from previous page

NaBr	-1.87	$2.07 \cdot 10^{-3}$	-1.84	$1.3 \cdot 10^{-3}$	–
NaCl	-2.13	$2.07 \cdot 10^{-3}$	-2.11	$9 \cdot 10^{-4}$	–
$NaCrO_2$	-2.27	$1.19 \cdot 10^{-2}$	-2.26	$2.88 \cdot 10^{-3}$	AFM
NaF	-2.98	–	-2.94	$1.3 \cdot 10^{-3}$	–
$NaFeO_2$	-1.81	$1.53 \cdot 10^{-2}$	-1.8	$2.72 \cdot 10^{-3}$	AFM
NaH	-0.29	$2.07 \cdot 10^{-3}$	-0.27	$6.5 \cdot 10^{-4}$	–
NaI	-1.49	$4.15 \cdot 10^{-3}$	-1.46	$2.75 \cdot 10^{-3}$	–
NaO_2	-0.9	$1.14 \cdot 10^{-2}$	-0.91	$5 \cdot 10^{-3}$	FM
NaS	-1.03	–	-1.08	$4.65 \cdot 10^{-3}$	–
$NaVO_3$	-2.47	$2.18 \cdot 10^{-2}$	-2.38	$1.75 \cdot 10^{-3}$	–
NiF_2	-2.27	$7.25 \cdot 10^{-3}$	-2.32	$3.97 \cdot 10^{-3}$	FM
NiO	-1.24	$6.74 \cdot 10^{-3}$	-1.22	$5.44 \cdot 10^{-3}$	AFM
P_2O_5	-2.23	$1.11 \cdot 10^{-2}$	-2.44	$1.43 \cdot 10^{-3}$	–
P_2S_3	-0.25	$2.18 \cdot 10^{-2}$	-0.46	$5.58 \cdot 10^{-3}$	–
P_3N_5	-0.41	–	-0.52	$5.81 \cdot 10^{-3}$	–
P_4S_3	-0.33	–	-0.36	$3.99 \cdot 10^{-3}$	–
PH_3O_4	-1.66	–	-1.88	$1 \cdot 10^{-3}$	–
Rb_2O	-1.16	–	-1.13	$6.67 \cdot 10^{-4}$	–
Rb_2S	-1.25	$7.22 \cdot 10^{-2}$	-1.16	$3.1 \cdot 10^{-3}$	–
RbBr	-2.04	$4.15 \cdot 10^{-3}$	-2.03	$1.3 \cdot 10^{-3}$	–
RbCl	-2.25	$4.15 \cdot 10^{-3}$	-2.24	$9 \cdot 10^{-4}$	–
RbF	-2.9	–	-2.78	$1.3 \cdot 10^{-3}$	–
RbH	-0.27	$2.07 \cdot 10^{-3}$	-0.24	$6.5 \cdot 10^{-4}$	–
RbI	-1.72	$2.59 \cdot 10^{-2}$	-1.7	$2.75 \cdot 10^{-3}$	–
$Si(NiO_2)_2$	-2.07	$1.36 \cdot 10^{-2}$	-2.11	$3.26 \cdot 10^{-3}$	FiM
$SrAl_2O_4$	-3.46	$2.47 \cdot 10^{-2}$	-3.46	$1.14 \cdot 10^{-3}$	–
$SrBr_2$	-2.48	$5.87 \cdot 10^{-3}$	-2.51	$1.73 \cdot 10^{-3}$	–
$SrCl_2$	-2.86	–	-2.89	$1.2 \cdot 10^{-3}$	–
SrF_2	-4.2	$1 \cdot 10^{-2}$	-4.23	$1.73 \cdot 10^{-3}$	–
SrH_2	-0.61	–	-0.65	$8.67 \cdot 10^{-4}$	–
SrI_2	-1.94	$7.25 \cdot 10^{-3}$	-1.96	$3.67 \cdot 10^{-3}$	–
$SrMoO_4$	-2.68	$1.73 \cdot 10^{-2}$	-2.67	$1.99 \cdot 10^{-3}$	–
SrO	-3.07	$1.97 \cdot 10^{-2}$	-3.08	$1 \cdot 10^{-3}$	–
SrO_2	-2.19	$5.22 \cdot 10^{-2}$	-2.21	$1.15 \cdot 10^{-2}$	–
SrS	-2.43	–	-2.4	$4.65 \cdot 10^{-3}$	–

Table S1 – continued from previous page

$TiCoO_3$	-2.5	$7.88 \cdot 10^{-3}$	-2.63	$1.7 \cdot 10^{-3}$	AFM
$TiFeO_3$	-2.57	$1.22 \cdot 10^{-2}$	-2.72	$2.35 \cdot 10^{-3}$	AFM
$TiMn_2O_4$	-2.59	–	-2.68	$1.9 \cdot 10^{-3}$	AFM
$TiMnO_3$	-2.82	–	-2.95	$1.6 \cdot 10^{-3}$	AFM
$TiNiO_3$	-2.49	$1.31 \cdot 10^{-2}$	-2.6	$2.45 \cdot 10^{-3}$	AFM
V_2O_3	-2.53	$3.52 \cdot 10^{-3}$	-2.53	$2.83 \cdot 10^{-3}$	FM
V_2O_5	-2.3	$2.52 \cdot 10^{-3}$	-2.29	$2.32 \cdot 10^{-3}$	–
VF_4	-2.91	$3.46 \cdot 10^{-2}$	-3.01	$2.44 \cdot 10^{-3}$	FM
VO	-2.24	$1.09 \cdot 10^{-2}$	-2.09	$3.35 \cdot 10^{-3}$	AFM
VO_2	-2.47	$3.45 \cdot 10^{-3}$	-2.48	$2.52 \cdot 10^{-3}$	FM
$W_{10}O_{29}$	-2.18	–	-2.17	$6.66 \cdot 10^{-3}$	FiM
WO_2	-2.04	$2.18 \cdot 10^{-2}$	-2.04	$8.54 \cdot 10^{-3}$	FM
WO_3	-2.18	$7.51 \cdot 10^{-3}$	-2.18	$6.5 \cdot 10^{-3}$	–
$Zn(FeO_2)_2$	-1.75	$8.74 \cdot 10^{-3}$	-1.75	$3.1 \cdot 10^{-3}$	AFM
$ZnCr_2O_4$	-2.29	$6.81 \cdot 10^{-3}$	-2.28	$3.29 \cdot 10^{-3}$	AFM

Table S2: Comparison of selected specie corrections with those calculated in literature.

Specie	Correction calculated in this work (eV/atom)	No. of compounds used in fit	Correction calculated by previous studies (eV/atom)	No. of compounds used in fit	Reference
oxide	-0.687	99	-0.68	6	[1]
oxide	-0.687	99	-0.599	16	[2]
N	-0.361	8	-0.446	7	[2]
F	-0.462	28	-0.442	14	[2]
Cl	-0.614	14	-0.483	10	[2]
Fe	-2.256	17	-1.723	3	[3]
Ni	-2.541	6	-2.164	1	[3]
Mo	-3.202	14	-2.668	2	[3]
V	-1.700	13	-1.764	4	[3]
Cr	-1.999	14	-2.067	2	[3]
Mn	-1.668	10	-1.687	4	[3]
Co	-1.638	8	-1.751	2	[3]

Table S3: Calibrated Hubbard U values used in the Materials Project[1]

Specie	U value (eV)
Co	3.32
Cr	3.70
Fe	5.30
Mn	3.90
Mo	4.38
Ni	6.20
V	3.25
W	6.20

[1]See `https://docs.materialsproject.org/methodology/gga-plus-u/`

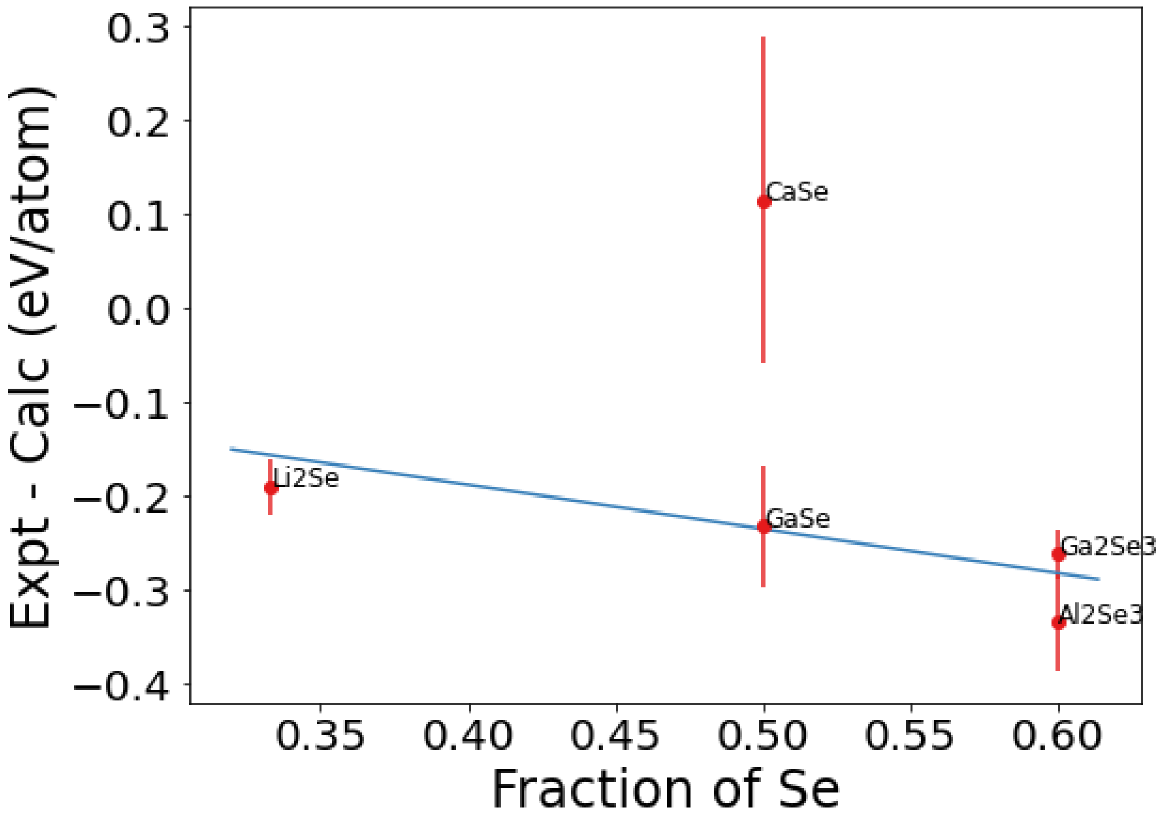

Figure S1: Errors in formation energy, δE, vs. fraction of Se for compounds used to fit the Se correction. Among these compounds, Se is the only atom being corrected, therefore δE should be a linear function of Se composition assuming a specie cross-wise independent, linear correction scheme. Note that compound CaSe only exhibits a small influence on the fitted correction (corresponding to the slope of the line) due to its large experimental uncertainty (see main text).

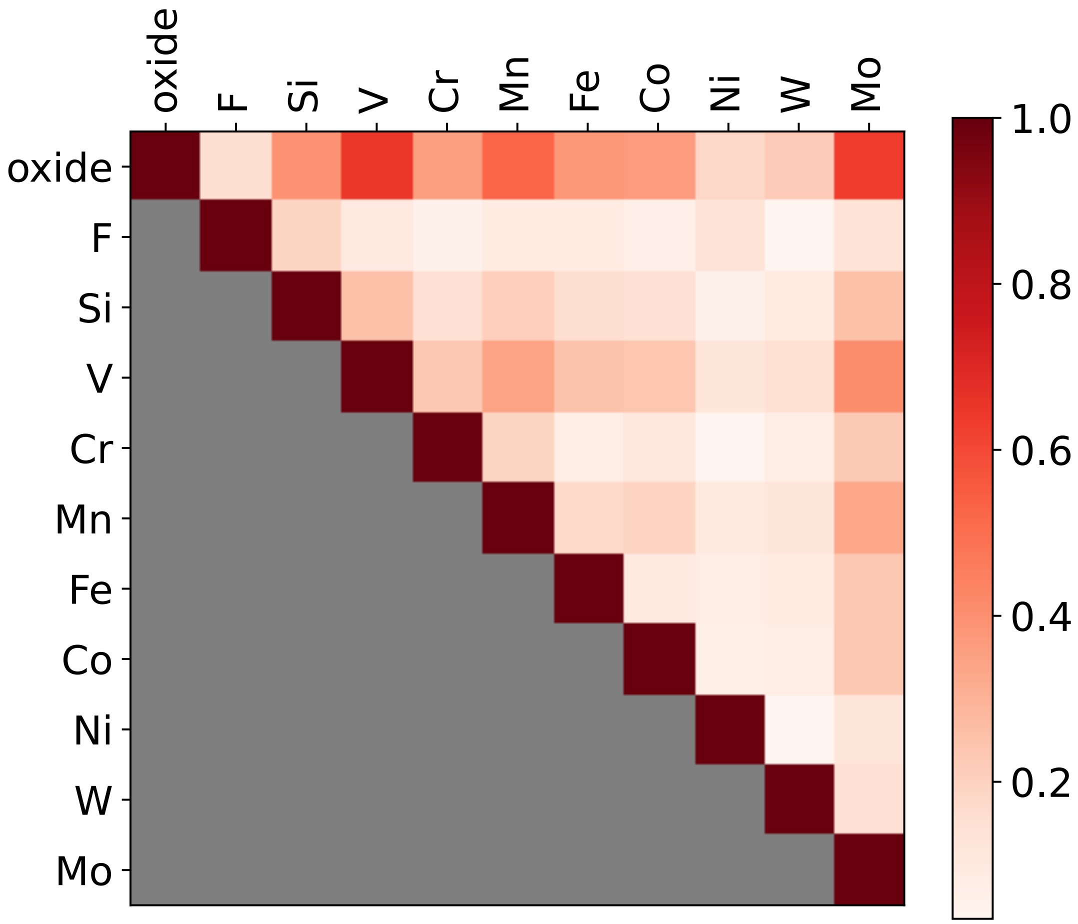

Figure S2: Covariance of the fitted corrections. Off-diagonal elements represent cross-correlation effects between species. Covariances are scaled to the value of the respective diagonal elements, which represent the variance of the individual specie corrections. Species with corrections whose covariance was always zero are not shown.

References

[1] Wang, L., Maxisch, T. & Ceder, G. Oxidation energies of transition metal oxides within the GGA+U framework. *Physical Review B - Condensed Matter and Materials Physics* **73**, 1–6 (2006).

[2] Grindy, S., Meredig, B., Kirklin, S., Saal, J. E. & Wolverton, C. Approaching chemical accuracy with density functional calculations: Diatomic energy corrections. *Physical Review B - Condensed Matter and Materials Physics* **87**, 1–8 (2013).

[3] Jain, A. *et al.* Formation enthalpies by mixing GGA and GGA + U calculations. *Physical Review B - Condensed Matter and Materials Physics* **84**, 1–10 (2011).
